# A Role of Inflammation in Charcot–Marie–Tooth Disorders—In a Perspective of Treatment?

**DOI:** 10.3390/ijms26010015

**Published:** 2024-12-24

**Authors:** Joanna Kamińska, Andrzej Kochański

**Affiliations:** 1 Institute of Biochemistry and Biophysics Polish Academy of Sciences, 02-106 Warsaw, Poland; kaminska@ibb.waw.pl; 2 Neuromuscular Unit, Mossakowski Medical Research Institute Polish Academy of Sciences, 02-106 Warsaw, Poland

**Keywords:** Charcot–Marie–Tooth disorders (CMTs), inflammatory neuropathies, immunomodulation, therapy

## Abstract

Despite the fact that there are published case reports and model work providing evidence of inflammation in Charcot–Marie–Tooth disorders (CMTs), in clinical practice, CMT and inflammatory neuropathies are always classified as two separate groups of disorders. This sharp separation of chronic neuropathies into two groups has serious clinical implications. As a consequence, the patients harboring CMT mutations are practically excluded from pharmacological anti-inflammatory treatments. In this review, we present that neuropathological studies of peripheral nerves taken from some patients representing familial aggregation of CMTs revealed the presence of inflammation within the nerves. This shows that neurodegeneration resulting from germline mutations and the inflammatory process are not mutually exclusive. We also point to reports demonstrating that, at the clinical level, a positive response to anti-inflammatory therapy was observed in some patients diagnosed with CMTs, confirming the role of the inflammatory component in CMT. We narrowed a group of more than 100 genes whose mutations were found in CMT-affected patients to the seven most common (*MPZ*, *PMP22*, *GJB1*, *SEPT9*, *LITAF*, *FIG4*, and *GDAP1*) as being linked to the coexistence of hereditary and inflammatory neuropathy. We listed studies of mouse models supporting the idea of the presence of an inflammatory process in some CMTs and studies demonstrating at the cellular level the presence of an inflammatory response. In the following, we discuss the possible molecular basis of some neuropathies involving neurodegenerative and inflammatory processes at both the clinical and morphological levels. Finally, we discuss the prospect of a therapeutic approach using immunomodulation in some patients affected by CMTs.

## 1. Introduction

Hereditary motor and sensory neuropathies (HMSN) known also as Charcot–Marie–Tooth disorders (CMT;) belong to the group of the most frequent neuromuscular disorders with a prevalence ratio estimated from 10 per 100,000 in Serbia to 82 per 100,000 inhabitants in Norway [1] with the worldwide frequency estimated to be 1:2500 [2]. At the clinical level, CMT disorders are characterized by a slowly progressive or even stationary course in which weakness and wasting of the distal muscles of the lower and upper limbs are the main symptoms. Many CMT-affected patients develop skeletal deformities such as scoliosis and pes cavus. Usually, CMT-affected patients remain ambulant, but in some especially recessive forms, they have to use wheelchairs. Usually, CMT is characterized by the slowly progressive course, but in some subtypes (hereditary neuropathy with liability to pressure palsies—HNPP, or hereditary neuralgic amyotrophy—HNA) neuropathy manifests with episodic attacks of muscle wasting. Some patients manifest with rare symptoms like optic nerve atrophy, learning difficulties, or vocal cord paresis [3].

To the best of our knowledge, no effective therapy was proposed for CMT diseases [4,5,6]. Although, in contrast to the 1990–2010 period in which the main discoveries in CMT genetics were made, at present, CMT studies have started to be focused on modeling the neuropathies and clinical trials in CMTs [7]. There are also data in the literature that, at least in some patients, show acute worsening of CMT course is associated with the superimposed inflammatory process, which accompanies hereditary neuropathy [8]. In recent studies, the question concerning the role of inflammatory processes in hereditary neuropathies has been addressed by numerous studies dedicated to the molecular pathogenesis of CMT. The deep understanding of the role of inflammation in the pathogenesis of CMT is important, because it may be directly translated to the therapy of this group of so-far incurable disorders.

## 2. The Presence of Inflammatory Process in the Patients Affected by CMT

The sharp dichotomy between hereditary and acquired (inflammatory) processes in peripheral neuropathies by means of pathophysiology was questioned by the detection of patients suffering from atypical pathology by means of a positive response to the anti-inflammatory treatment [9]. Since reports of the co-occurrence of inflammatory and hereditary processes were very sporadic and limited to single families or even occasional patients, the question arose as to whether this was a matter of accidental coincidence rather than a real combination of the two processes resulting from the presence of a single pathogenic mutation. Moreover, so far, the molecular diagnosis of CMT has been limited to the relatively arbitrarily selected genes from the list of more than 100 proteins. Thus, there was still a real possibility that the sequence variants identified in this pre-selection-based process did not play any causative role in the pathogenesis of hereditary/inflammatory neuropathy. Nowadays, the presence of inflammation features in CMT-affected patients has started to be confirmed in studies of large cohorts of patients screened for mutations in hundreds of genes. The role of the Next-Generation Sequencing technology has proved especially promising and effective in patients affected by heterogeneous diseases, like CMT, which are caused by mutations in one of more than 100 genes. On the other hand, with the automatization of this technology, the groups of the examined patients could be expanded, which was similar to a large multicenter European study, in which more than 1000 patients suffering from chronic inflammatory demyelinating polyneuropathy (CIDP) were analyzed using a CMT gene panel covering 120 genes. Interestingly, mutations within CMT genes were found in a group of 35 patients with CIDP, which accounts for 3.5% of cases. The vast majority of mutations were located within the *MPZ* gene (11), followed by the *PMP22* and *GJB1* genes. Single mutations were found in the *AARS*, *BSCL2*, *LRSAM*, *MFN2*, *NEFH*, *NEFL*, *PLEKHG1*, *SH3TC2*, and *YARS* genes [10]. In a similar study conducted in four children affected with CIDP, a genetic analysis based on a study of 70 CMT-associated genes identified mutations in the *SH3TC2*, *MPZ*, and *PMP22* genes. The patients with these mutations manifested with neuropathy with subacute onset typical for inflammatory neuropathies, but there was no convincing response to intravenous immunoglobulin therapy. This study shows that the distinction between the hereditary and inflammatory processes may be challenging even for experienced neurologists [11]. Based on these data, at least mutations in the *MPZ*, *PMP22*, *GJB1*, and *SH3TC2* genes could be considered as causative for both processes, i.e., inflammation and genetics-based pathology. There are supporting examples of this idea. First is the case of a 31-year-old woman with symptoms of stable hereditary neuropathy caused by a mutation in the *MPZ* gene resulting in Ser63del. The patient experienced episodes of rapid worsening of the clinical course of the neuropathy, which responded to corticosteroid treatment [8]. The other example comes from the recent study by Chahbouni and colleagues who reported normalization of pro-inflammatory cytokines in CMT1A-affected children treated with melatonin [12]. The coexistence of hereditary CMT1A and inflammatory neuropathy was also described in seven patients. Of this group, four patients were treated with steroids and/or intravenous immunoglobulin, and three of them responded positively to treatment [13]. In another case, a 15-year-old girl was diagnosed with acute inflammatory demyelinating neuropathy and went through plasmapheresis. After this treatment, some of her conditions were improved, but the presence of pes cavus and findings from biopsies and electrophysiological examinations were inconsistent with the primary diagnosis. Further genetic analysis revealed a duplication involving the *PMP22* gene in her and the two family members included in this study [14]. Interestingly, in a series of patients harboring the recurrent and homozygous mutation in the *SH3TC2* gene, resulting in Arg954X, one patient was shown to have a 20-year history of superimposed inflammatory neuropathy [15]. In CMTX1 disease caused by mutations in the *GJB1* gene encoding connexin 32 (Cx32), some patients also manifest with additional symptoms due to transient involvement in the Central Nervous System (CNS) [16]. Magnetic resonance imaging (MRI) studies show typical multiple sclerosis lesions in CMTX1-affected patients with CNS involvement. These lesions were transient and disappeared after 2–3 months from the attack of CMTX1 [16]. Another example is a patient with pain and numbness in an arm who responded positively to intravenous immunoglobulin treatment and was found to have a *GJB1* gene mutation resulting in Cys173Arg substitution in Cx32 [13]. In agreement with the previous findings, a 22-year-old patient manifesting with CNS involvement, harboring substitution of Ser26Leu in Cx32, showed a positive response to the treatment with methylprednisolone and natalizumab (a glucocorticosteroid with anti-inflammatory and immunosuppressive effects and monoclonal antibody, respectively), normally used in the treatment of the patients with multiple sclerosis [17].

The presence of inflammatory processes in CMT-affected patients was not only reported for *MPZ*, *PMP22*, *GJB1*, and *SH3TC2* genes but was also identified in patients suffering from rare subtypes of CMT. A good illustration of the inflammatory processes present in rare CMT subtypes seems to be the case of a 26-year-old man with the rapid development of neuropathy over the period of 2 years and a marked fall in conduction velocity in the median nerve over 6 years who was shown to harbor a mutation in the *FIG4* gene. However, the clinical and electrophysiological picture observed in this patient was consistent with inflammatory neuropathy, but no features of active inflammation were detected in the sural nerve biopsy [18]. Also, in a group of eight patients harboring the same mutation in the *FIG4* gene (Ile41Thr), two patients manifested with the phenotype resembling inflammatory neuropathy were reported [19].

In other rare subtypes of CMT, i.e., the CMT1C disease caused by mutations in the *LITAF* gene, atypical phenotypes resembling acquired inflammatory neuropathy have also been reported. A 56-year-old woman manifesting with rapidly progressing weakness of a single upper limb partially responsive to immunotherapy was shown to carry a pathogenic mutation within the *LITAF* gene [20]. In a study of a large cohort of 1143 CMT Korean families, only in three families was a mutation in the *LITAF* gene resulting in Gly112Ser substitution identified. In 22% of CMT1C-affected patients, a conduction block typical for acquired inflammatory neuropathy was found [21].

The coexistence of primary progressive multiple sclerosis (PPMS) and CMT1C disease may not result from the presence of the mutation in the *LITAF* gene, but the role of mutated LITAF in inflammation (PPMS) cannot be definitively excluded [22].

At a clinical level, the question can be asked regarding the inverse relationship; i.e., How often do patients affected by CMT manifest overt or subclinical features of inflammatory neuropathy? In other words, can it be considered a reasonable approach to treat some CMT-affected patients with certain anti-inflammatory drugs? Is it reasonable to test the effect of anti-inflammatory drugs on improving at least the electrophysiological features of polyneuropathy? Is it reasonable to check the impact of anti-inflammatory drugs on the improvement of at least the electrophysiological features of polyneuropathy? And moreover, does there exist any available biomarker of the inflammatory process suggesting the activation of inflammation in CMT-affected patients? Is it possible to use the elevated protein level in cerebrospinal fluid as an applicable biomarker of the inflammation process in CMT-affected patients? Or maybe a better approach would be to use electrophysiological parameters indicative of changes in the clinical course of neuropathy [12]? And on the other side, should the patients suffering from inflammatory neuropathy be checked for mutations at least in the *MPZ*, *PMP22*, or *SH3TC2* genes?

## 3. The Role of Inflammation Process in the Mice Models of CMT

Due to the obvious limits of experimental studies in CMT-affected patients, the role of immune cells in the pathogenesis of CMT cannot be precisely established without suitable animal models of CMT. Several different rodent models of CMT1A disease exist, which more or less accurately reflect the pathological changes and course of the disease [23]. Gene therapy studies using one of these models, the C61-het mouse model, showed an accumulation in the peripheral nervous system of inflammatory infiltrates that increase with age. The decrease in the number of immune-competent cells in the sciatic nerve was observed as an effect of gene therapy [24]. Mice heterozygously deficient in *MPZ* gene expression (coding P0 protein) may serve as a good model of human CMT1B disease caused by mutations in the homologous human *MPZ* gene. Till the age of 4 months, the *MPZ*+/− heterozygous mice do not develop demyelination of peripheral nerves. The process of demyelination is accompanied by the presence of CD8-positive T-lymphocytes residing in the endoneurium [25,26,27,28]. Interestingly the number of infiltrating immune cells increases with the process of demyelination. The question of whether immune cells react only to the genetically induced process of demyelination or take part in the wider process of demyelination was answered by the cross-breeding experiments between the *MPZ*+/− and mice deficient in B- and T-lymphocytes. Thus, mice lacking the recombination activating gene 1 (*RAG-1*) were crossed with P0+/− mice. As a result, it was found that they did not develop as severe demyelinating neuropathy as *MPZ*+/− mice [27]. The process of demyelination in double-mutant (*MPZ+/− RAG-1−/−*) mice has been shown to be reversible. The bone marrow transplantation from wild-type mice to double-mutant (*MPZ+/−*; *RAG-1−/−*) mice resulted in the aggravation of the demyelination process [29]. In other words, the T- and B-lymphocytes and wider immune processes possess the ability to modulate the process of demyelination at least in the mice model of CMT1B disease. The T- and B-lymphocytes are not the only immune cells that have been observed in the endoneurium of the *MPZ*+/− mice. Macrophages have also been observed beginning from the age of 6 months in the peripheral nerves of *MPZ*+/− mice. The leading role of macrophages in the process of demyelination in this model was proved by experiments in which the *MPZ*+/− mouse was crossed with the mouse deficient in macrophage colony-stimulating factor (M-CSF) [*op*/*op*]. The obtained double [*MPZ*+/−; *op*/*op*] mutants manifested with less severe demyelination than *MPZ*+/− mice. The double mutants showed more fibers with a thicker myelin sheath than *MPZ*+/− mutants. Given that the M-CSF receptor is present exclusively on the macrophages, the impaired macrophage activation process may directly be associated with demyelination at least in the *MPZ*+/−; *op*/*op* mice model [30]

The role of immune cells is not limited to CMT1B disease and its animal models. The presence of inflammatory processes was also demonstrated in the mice model of CMTX1 disease. *GJB1*-deficient mice develop progressive demyelinating neuropathy. Similar to *MPZ*+/−mice, *GJB1*-deficient mice presented the elevation of macrophages within demyelinated nerves [31,32,33]. Macrophages have been found in the quadriceps and saphenous nerves of 3-, 6-, and 12-month-old *GJB1*-deficient mice. The immunoelectron microscopy images showed macrophages penetrating the basal lamina of Schwann cells, contacting partially vacuolized myelin. Similarly to the *MPZ*+/− mice, a significantly elevated number of CD8-positive lymphocytes was reported in the *GJB1*-deficient mice [31]. Some mutations of the *GJB1* gene have been shown to aggravate the process of inflammation. In an experimental model of inflammation induced by the injection of lipopolysaccharides (LPSs) in mice harboring a mutation in the *GJB1* gene resulting in Tyr55Ile substitution in Cx32, widespread inflammation involving CNS was reported. Interestingly *GJB1* knock-out mice did not develop as severe an inflammatory response as those with Tyr55Ile substitution after LPS injection. The range of inflammation in *GJB1* mutant mice correlated with the level of endoplasmic reticulum (ER) stress resulting from misfolded protein [34].

Both clinical observations made in CMT1A, CMT1B, and CMTX1 patients and morphological findings observed in the animal models of these diseases indicate that immune cells play a leading role in the process of neurodegeneration at least in the hereditary demyelinating neuropathies. In addition to the experiments described above, other experiments have been carried out to draw similar conclusions. The effect of macrophage colony-stimulating factor (CSFI) in CMT1B and CMTX1 disease pathology was confirmed in an elegant study. In this study, pharmacological blockade of the CSFI receptor (CSFIR) resulted in an improved phenotype in mice with CMT1B and CMTX1. Neuropathy in the mouse models of these diseases was shown to be ameliorated at three levels, i.e., morphological, electrophysiological, and clinical (grip strength testing) [35]. These findings raise the question of the underlying mechanism leading to this inflammation. In line with the above-described role of the CSFI receptor, CSF1-1 deficiency was shown to lead to the amelioration of neuropathy in the peripheral nerves of *GJB1*-deficient mice. The other studies showed also that the loss of connexins Cx47 or Cx32 in mice results in the dysregulated expression of cytokine genes [36]. Also, in human biopsies obtained from patients with CMT1X, the role of CSF1-1 was documented [37]. The role of the inflammatory process in CMT was also documented in two works by Kagiava et al., who showed that gene therapy performed on *Gjb1* null mice reduces inflammation in peripheral nerve tissues [38,39]. In the case of CMT4 caused by recessive mutations in *GDAP1*, neuroinflammation could also play a role in the pathology of neuropathy, as suggested by a study of *GDAP1*−/− mice in which the transcriptome analysis of the spinal cord revealed the deregulation of genes of innate and adaptive immune response pathways and the presence of inflammation process, localized particularly to the spinal cord and peritoneum [40]. However, the morphological observations of the inflammatory process in CMT have been mainly focused on CMT1A, CMT1B, CMTX1, and CMT4 diseases, but may indicate, at least in general, an important role for inflammation in hereditary peripheral neuropathies. These traditionally morphological studies in CMT played an important role in the diagnosis of hereditary neuropathies, but they are now gradually being replaced by investigations on the cellular and molecular level. The data from paragraph 2 and 3 are summarized in Table 1.

## 4. The Molecular Changes That Could Lead to Inflammation in Selected CMT Types

The examples described above for the involvement of the inflammation process in the course of diseases caused by mutations in the *MPZ* and *GJB1* genes point to the activation of innate immunity and the mobilization of adaptive (lymphocytic) immunity to antigens present in the affected tissue. However, nothing is known about the molecular mechanisms underlying inflammation in the examples described.

**Figure 1:**
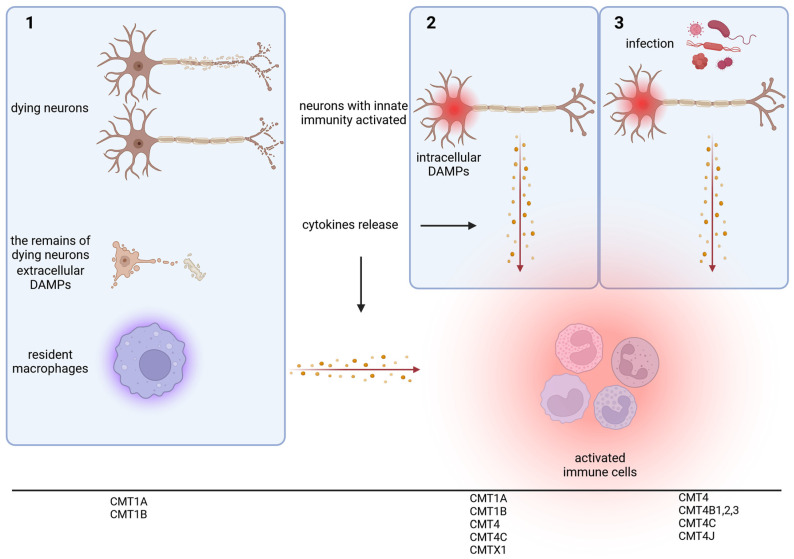
Three scenarios for the development of inflammation in CMT: mutation leads to cell death, and residual cells activate the immune system (**1**); mutation results in activation of the innate immune response (**2**); pathogen-activated immune response is not effectively silenced (**3**). The association of these scenarios to different subtypes of CMT is given. Created in BioRender.

At least three scenarios can be considered (Figure 1). The first assumes that cells die because their molecular mechanisms are unable to compensate for the changes caused by CMT gene mutations and cell debris activates resident macrophages. This leads also to the release of damage-associated molecular patterns (DAMPs). DAMPs are recognized by pattern recognition receptors on immune cells. This triggers a cascade of cellular events leading to the production of pro-inflammatory signals. Such an inflammation caused by dying cells can lead to damage to surrounding “healthy” tissue.

The second scenario is based on the fact that the innate immune response can be induced in almost all types of cells malfunctioning due to mutations, producing intracellular DAMPs. Such a DAMP can be, for example, one’s own DNA, which is recognized by cGAS (cyclic GMP-AMP synthase), resulting in the activation of the cGAS-STING pathway. This pathway is normally responsible for recognizing foreign (bacterial, viral) DNA. As a consequence of such activation, cells release proinflammatory factors themselves. For example, it is known that after sciatic nerve injury, motoneurons activate the stimulus-induced cytoplasmic multimeric protein complexes called inflammasomes [41]. This complex, in response to the presence of DAMPs, activates procaspase-1 to form caspase 1 and induces cleavage and the secretion of proinflammatory cytokines to activate an acquired response.

The third scenario is that inflammation occurs as a result of infection, but due to mutations in CMT genes that encode proteins involved in processes important for silencing the immune response, this silencing does not occur or is slowed down. This can result in chronic inflammation indirectly caused by the mutation.

The scenario in which nerve cells per se induce inflammation is probable in the case when mutations cause the retention of mutant protein in ER. This is in the case of some *GJB1*, *PMP22*, or *MPZ* mutations. The *GJB1* gene encodes connexin32 (Cx32). Cx32 participates in the form of trans-myelin gap junctions in Schwann cells. *PMP22-* and *MPZ*-encoded proteins (Pmp22 and P0, respectively) are components of myelin. All three proteins are transported through the ER and Golgi apparatus to the plasma membrane. Some Cx32, PMP22, or P0 mutant proteins are retained in the ER [42,43,44], which may result in the activation of an unfolded protein response (UPR). The UPR response is associated with activation of the transcription factor NF-κB (nuclear factor kappa-light-chain-enhancer of activated B cells) [45,46]. This factor induces the expression of various proinflammatory genes and is thus involved in the innate and adaptive immune response. There is also another possibility that ER overload per se, even if the proteins are normally folded, may result in NF-kB activation via the non-classical UPR pathway, which is dependent on calcium ions (Ca^2+^) and reactive oxygen species (ROS) [47]. These scenarios remain to be verified experimentally. The induction of inflammation cannot be ruled out when there are mutations in the *GJB1*, *PMP22*, or *MPZ* genes, which do not alter the localization of the proteins they encode or cause them to accumulate in the Golgi apparatus. This must occur by a different mechanism. In cases where mutant Pmp22 and P0 proteins are incorporated into myelin, or where there is an excess of Pmp22, leading to changes in the myelin sheath and its subsequent breakdown, inflammation can arise according to the first proposed scenario, i.e., due to cell death.

In the case of *SH3TC2* gene mutation-caused disease, the inflammation described in the patient can be evoked based on any of the proposed scenarios. The SH3TC2 protein is a recycling endosome protein and effector of the small GTPase Rab11. Importantly, all disease-causative mutations in *SH3TC2* result in SH3TC2 protein variants being unable to associate with Rab11 and consequently lose their localization in recycling endosomes [48]. On the other hand, the SH3TC2 protein interacts with one of the integrin isoforms, which is necessary for the proper myelination of peripheral nerve axons by Schwann cells [49]. Thus, SH3TC2 mislocalization will result in changes in integrin trafficking and subsequent myelin formation, which can trigger inflammation. Interestingly, as *SH3TC2* gene mutations in patients can lead to inflammation [15], the lack of the RAB11a protein, studied in mouse colon cells, resulted in the presence of neutrophils. Also, the production of inflammatory cytokines and activation of NF-κB in epithelial cells was detected [50]. This suggests that it is worth checking if, in cells with a mutation in *SH3TC2*, preferably derived from patients, NF-κB is activated.

For termination of inflammation signaling (for example after pathogen infection, the third scenario), the efficient degradation of elements of activated inflammation pathway components in lysosomes is necessary. This degradation requires vesicular trafficking like the endocytic pathway and Golgi apparatus to endosome and lysosome pathways to operate properly, and blocking at any step of these multistep pathways will result in problems with silencing of the inflammation both innate and induced. It was shown that cGAS-STING signaling termination is based on STING degradation in lysosomes [51,52]. The ubiquitinated STING is recognized on endosomes by subunits of ESCRT (Endosomal Sorting Complex Required for Transport) complexes, and a lack of these complexes enhances STING signaling [53]. ESCRT complexes recruitment to endolysosomal membranes and thus their function is regulated by phosphatidylinositols (PIPs). PIPs are not abundant, but essential lipids and their presence and level in membranes are tightly regulated by lipid kinases and phosphatases. Importantly these lipids are specific to particular membranes where they participate in the regulation of various events, such as transport vesicle formation, membrane fusion, and dynamics. This is because PIPs play a role in the recruitment and/or activation of their effector proteins. The PIP specific for endosomes and lysosomes is phosphatidylinositol 3 phosphate (PI3P), which is converted to PI(3,5)P_2_ by PIKfyve lipid kinase. The PI(3,5)P_2_ could be further dephosphorylated by Fig4 and myotubularin or myotubularin-related phosphatases to PI3P or PI5P, respectively [54].

Interestingly, some mutations in the *FIG4* gene led to CMT type 4J disease, a recessive neuromuscular disease characterized by abnormal lysosomal storage, and segmental demyelination in humans [55]. A truncation of Fig4 due to a mutation that is produced through transposon insertion results in a 40–71% reduction in fibroblast PI(3,5)P_2_ levels [56,57] and leads to deficient sensory neurons in which “bubble-like” vacuoles, being dilated endolysosomes, are observed. These vacuoles are localized in perinuclear regions and interfere with intracellular organelle trafficking processes [58,59]. Similarly, in Fig4, deficient cortical/spinal motor neurons and glia, the dysfunctional lysosomes accumulating abnormally large quantities of lipids and proteins, were described. These cellular phenotypes are reminiscent of those of other lysosomal storage disorders (LSDs) such as Niemann Pick C, Tay-Sachs, and Mucolipidosis type IV. In agreement, newer data suggest that the main problem caused by the *FIG4* mutation is not connected with phosphoinositide metabolism but with a lack of lysosomal membrane homeostasis. This is in line with the very recent finding that lysosomal storage diseases (LSDs), which are characterized by the genetically determined metabolic dysfunction of lysosomes, strongly autoactivate neuronal cGAS-STING signaling, leading to neuronal death and disease progression. This type of signaling is triggered by the presence of dsDNA in the cytoplasm. This suggests that if the anti-inflammatory treatment strategy proposed for LSD proves to be effective, it may also be applicable in CMT when the molecular picture indicates lysosome dysfunction [60].

Similarly, mutations in the gene encoding myotubularin MTM1 or the MTMR2, MTMR5 and MTMR13 genes for myotubularin-related phosphatases cause neuromuscular diseases: X-chromosome-associated myotubular myopathy (XLMTM also called centronuclear myopathy) and CMT types: CMT4B1, CMT4B3 and CMT4B2, respectively [54], and in the case of mutations of the *VAC14* gene, which encodes a scaffold protein for the PIKfyve kinase complex, result in progressive neurological disorders [61].

Interestingly, it was also shown recently that in *GDAP1^−/−^* mutant mice, devoid of the ganglioside-induced differentiation-associated protein 1 (GDAP1) encoding gene, the level of PI3P is reduced. Moreover, cells have enlarged lysosomes similar to what is observed when the level of PI(3,5)P_2_ is reduced and the GDAP1 protein itself interacts with PIKfyve kinase [62]. Moreover, Western blot analysis showed an increased level of pro-inflammatory proteins, excluding tumor necrosis factor-alpha (TNF-α), and an increase in inflammatory proteins of the innate immune system in the spinal cord and sciatic nerve [40]. Also, motor neurons differentiated from *GDAP1^−/−^* iPSCs have an increase in innate immune response markers [63].

In the case of mutations in the *GDAP1* gene, the inflammation can be triggered not only by changes in levels of some PIPs but also by changes in mitochondria homeostasis and by increased levels of ROS, which are known to trigger inflammation. The observation that in HeLa cells expressing mutated dominant alleles of *GDAP1*, the organization of the trans-Golgi network (TGN) is changed as visualized by the mislocalization of a marker protein, TGN46 [64], and changes in its modification further support the view that inflammation signaling is induced by mutations in *GDAP1*. The dispersion of TGN is observed in cells with activated inflammasome NLRP3 [65].

Indeed, in regard to CMT diseases, it was also shown that mutations in the *LITAF/SIMPLE* gene encoding LPS-Induced TNF-Alpha Factor (LITAF) are linked to CMT1C, a demyelinating peripheral neuropathy. Interestingly, LITAF is a moonlighting protein. Although originally it was found as a subunit of a complex responsible for the up-regulation of the transcription of different cytokines encoding genes [66,67], later studies showed its localization in endosomes, multivesicular bodies (MVBs), and lysosomes, where it interacts with the ubiquitin ligases of the NEDD4 (Neural Precursor Cell Expressed, Developmentally Down-Regulated 4) family and with the subunits of ESCRT complex subunits: Hrs1 (hepatocyte growth factor regulated kinase substrate) or TSG101 proteins (Tumor Susceptibility 101). Moreover, deletion of *LITAF* reduces the formation of MVBs in mice primary embryonic fibroblasts [68], and one of the amino acid residue substitutions found in patients results in altered changes in Hrs1 localization [69]. Importantly, the improper formation of MVBs in CMT1C patient B cells and abnormal vacuolization in the patient’s fibroblasts were also observed [69,70]. Although, it was shown that the CMT1C-associated mutations in *LITAF* neither affected the interaction of LITAF with NEDD4 or TSG101, nor did they lead to its altered subcellular localization [71] in 293T cells, but in another experimental model, it was demonstrated that the same mutations caused the release of LITAF to cytoplasm or its mistargeting to mitochondria. Interestingly, patients harboring *LITAF* gene mutations do not always develop disease. It is reported that the additional mutation in the *EGR2* gene, the encoding transcription factor, is necessary to cause CMT disease [72]. Similarly, the effect of amino acid residue Val144Met substitution in LITAF is modified by polymorphisms in the promoter region of the TNF-α gene causing the development of primary progressive multiple sclerosis [22]. Nevertheless, it has been demonstrated that at least LITAF Thr115Asn substitution changes the inflammatory signaling kinetics in a mice model [69], and further experiments should address whether this is due to other CMTs causing mutations in *LITAF*.

Based on the data presented above, showing the links between changes in cell function and inflammation, it seems that virtually any change can lead to the induction of inflammation. Therefore, it is important to study the molecular mechanisms of disease to determine the contribution of inflammation to the pathogenesis of diseases, including CMT diseases as in the cases described. This research will not only contribute to the advancement of knowledge, but it can be used to propose a target for therapeutic intervention.

## 5. A Perspective on Anti-Inflammatory Treatment in CMTs—Which Patients Should Be Treated and When?

In the previous paragraphs, we have shown that the inflammatory process in CMT was documented at four levels, i.e., clinical, electrophysiological, morphological, and finally, molecular for several types of CMT. With this knowledge, it seems reasonable to ask the question, how these observations may be used to monitor (potential biomarkers) inflammatory processes in CMT. Only when the inflammatory process in CMT starts to be measurable can we consider the effectiveness of anti-inflammatory treatment in CMT-affected patients.

Despite numerous efforts to treat Charcot–Marie–Tooth (CMT) disorders in recent years, an effective pharmacological approach has not been established even in the most common subtype of neuropathy, i.e., CMT1A disease caused by the duplication of the *PMP22* gene [5].

In this context, the possibility of modulation of the CMT clinical course with anti-inflammatory treatment seems to be very promising. Anti-inflammatory treatment could also be used as a synergistic complement to the applied treatment. The key question concerns the moment at which CMT should be treated with anti-inflammatory drugs. The second question addresses the subtypes of CMT, which should be considered in the decision of anti-inflammatory treatment. It seems too early to establish a complete list of CMT mutations “prone” to anti-inflammatory treatment. The only large study encompassing 1000 patients with chronic inflammatory demyelinating neuropathy (CIDP) screened for CMT mutations seems to be merely a good example or, indeed, a pilot study for further analysis [10].

On the other hand, many more important studies reporting meaningful statistics on the percentage of inflammatory components within certain subtypes of CMT disorders are lacking. Finally, we have no efficient biomarker indicating the presence of inflammatory processes in CMT, which could detect the inflammatory component and, later on, monitor the effectiveness of the anti-inflammatory treatment.

Although the observation of a rapid deterioration of the clinical course in CMT-affected patients supported by electroneurography (ENG) results may be considered as evidence of an inflammatory process, ENG parameters do not appear to be specific and sufficient enough. Moreover, without access to a specific biomarker of inflammation at the cellular or even at the molecular level, the vast majority of patients suffering from CMT with subclinical inflammatory neuropathy may be missed or not included in the therapy. As long as the molecular genetic results obtained in the CMT-affected patients are not accompanied by an analysis of cellular biomarkers of inflammation, we will not be able to estimate the real role of inflammatory processes within CMT. In fact, we still do not know the role of the inflammatory processes in CMT in the intra- and interfamilial variability of the clinical picture of certain neuropathy. The clinical variability of CMT may also result from the coexistence of a leading driver mutation with mild/weak CMT sequence variants (genetic background) [73]. We cannot exclude the possibility that an inflammatory component may contribute to the heterogeneity in the pattern of the effects of the CMT gene mutations.

In recent years, due to the implementation of CMT gene panels covering hundreds of CMT genes, clinical variability within families was at least partially elucidated by the additive effect of two mutations in CMT genes, so-called concomitant variants. The frequency of concomitant variants in CMT resulting in significant intrafamilial clinical variability seems to be not rare. In a relatively large cohort of 189 CMT-affected patients, concomitant variants were found in 2.1% of the total cohort [74].

At this moment, the clinically important question is raised, whether clinical intrafamilial variability results from the presence of two or even more concomitant variants in CMT genes or from inflammatory components? To differentiate these two processes, the first step should be the analysis of the panel of 120 CMT genes instead of single-gene analysis.

Moreover, in general, the inflammatory component will be rather characterized by the rapid worsening of the clinical course of CMT in contrast to patients harboring concomitant mutations and manifesting with a stable slowly progressive phenotype more severely expressed than in the remaining family members [75].

In other words, at least in some CMT-affected patients harboring the driver/major mutation in one of the CMT genes that are prone to inflammation, the genetic noise/background of weak sequence variants should be analyzed. From the opposite side, given the rising access to massive gene sequencing, we may consider the question of whether patients suffering from typical inflammatory neuropathy should be screened for mutations in CMT genes.

Especially in patients manifesting with CMT and being negative for CMT mutations, anti-inflammatory treatment could be considered. In case of a positive response to anti-inflammatory treatment, should this kind of management be continued despite the clinical picture suggesting the diagnosis of hereditary neuropathy?

To conclude, the introduction of effective anti-inflammatory treatment, at least in some CMT-affected patients, seems to be beneficial. Unfortunately, up to the moment when any biomarker of inflammation will be available, the decision of the anti-inflammatory treatment must/has to be based on careful clinical observation accompanied by the results of neurographic studies.

## Figures and Tables

**Table 1:** Summary of the CMT subtypes in which the inflammatory process has been reported in patients or described in mouse models.

CMT Type	Mutated Gene	Protein	Inflammation in Patients	Inflammation in Mice Models
CMT1A	*PMP22*	PMP22 (Peripheral Myelin Protein 22)	[10,11,13,14]	[24]
CMT1B	*MPZ*	P0 (Myelin Protein Zero)	[8,10,11]	[25,26,30]
CMT1C	*LITAF*	LITAF (Lipopolysaccharide-Induced TNF Factor)	[20,21,22]	
CMTX1	*GJB1*	Cx32 (Connexin32)	[10,13,16]	[31,32,38]
CMT4	*GDAP1*	GDAP1 (Ganglioside Induced Differentiation Associated Protein 1)		[40]
CMT4C	*SH3TC2*	SH3TC2 (SH3 Domain And Tetratricopeptide Repeats 2)	[10,11,15,17]	
CMT4J	*FIG4*	FIG4	[18,19]	
